# Ag/H-ZIF-8 Nanocomposite as an Effective Antibacterial Agent Against Pathogenic Bacteria

**DOI:** 10.3390/nano9111579

**Published:** 2019-11-07

**Authors:** Yanmei Zhang, Xin Zhang, Jie Song, Liming Jin, Xiaotong Wang, Chunshan Quan

**Affiliations:** 1 College of Life Science, Dalian Minzu University, Economical and Technological Development Zone, Dalian 116600, China; zxin326326@163.com (X.Z.); m18340873502@163.com (X.W.); songjie_o@sina.com (J.S.); jlm@dlnu.edu.cn (L.J.); 2 Key Laboratory of Biotechnology and Bioresources Utilization, Dalian Minzu University, Ministry of Education, Dalian 116600, China

**Keywords:** Ag nanoparticles, hierarchical, ZIF-8, antibacterial activities, synergistic effect

## Abstract

Development of antimicrobial nanomaterials is one of the most attractive strategies for eliminating the major threat of pathogenic bacteria to public health. In this work, we developed a simple impregnation-reduction method for the synthesis of Ag-doped hierarchical ZIF-8 (Ag/H-ZIF-8) nanocomposite. The nanocomposite was characterized by several techniques and its antibacterial activity was investigated. Ag nanoparticles are uniformly dispersed in the porous ZIF-8 with narrow size distribution. Consequently, the resulting Ag/H-ZIF-8 nanocomposite showed significantly enhanced antibacterial activities compared to the single ZIF-8 or Ag nanoparticles. Furthermore, the composite is biocompatible, because no obvious toxicity was observed on Hepatic epithelial cells. This study offers a new approach for the design of hybrid antimicrobial nanomaterials that have great potentials in practical disinfections.

## 1. Introduction

In the past decades, bacterial infections have become a major threat to public health, especially with the abuse of antibiotics and chemical bactericides. Hence, considerable efforts have been devoted to the development of novel and effective antibacterial agents to control the pathogenic bacteria growth [1,2,3]. Recently, inorganic disinfectants have been becoming particularly attractive as alternatives to organic compounds because they are more stable, easier to produce and environmentally benign.

It is well known that nano-sized silver (Ag) nanoparticles (NPs) are excellent antimicrobial agents against both gram-negative and gram-positive bacteria [3,4,5]. However, nano-structured silver nanoparticles without surface functionalization/stabilization are thermodynamically unstable and are easily aggregated into large inactive particles [6]. Stabilizing NPs in the matrices of solid materials has been proved to be one of the most common and efficient strategies for protecting them against aggregations [7,8,9,10]. Among various reported solid materials, metal-organic frameworks (MOFs) have great potential owing to their highly porous structure, tunable surface properties, versatile framework functionalities and adjustable pore sizes [11]. In particularly, MOFs can be utilized as efficient supports for metal NPs with controllable size, because they can control the limited growth of metal NPs, providing a powerful confinement effect and preventing their aggregations [12,13]. 

**Scheme 1:**
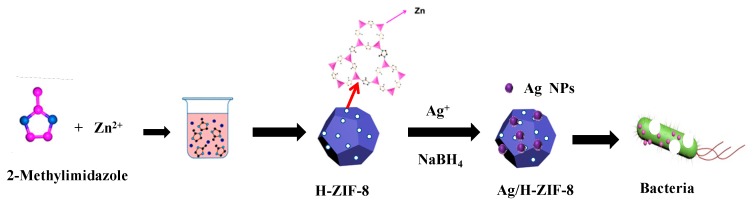
Fabrication of Ag/H-ZIF-8 composite and its antibacterial activity.

Bearing this in mind, as depicted in Scheme 1, hierarchical ZIF-8 was selected as support for stabilizing the metallic Ag NPs. Consequently, uniformly dispersed Ag Nps with narrow size distribution were produced in the hierarchical ZIF-8. The resulting Ag/H-ZIF-8 nanocomposite demonstrated enhanced antibacterial activity against both gram-positive and negative pathogenic bacteria strains than that of single ZIF-8 or Ag NPs, probably due to the synergistic effect between the Ag NPs and the hierarchical ZIF-8. The hybrid material presented here displays great potential in practical disinfection.

## 2. Experimental 

### 2.1. Materials

All the chemical reagents were purchased from commercial suppliers and used as received without further purification. Citric Acid (AR, 99.5%) was purchased from Tianjin Nankai Chemicals (Tianjin, China). L-histidine (His 99%) was obtained from Sangon Biotechnology Inc (Shanghai, China). Zn (NO_3_)_2_·6H_2_O (AR, 99%) and triethylamine (TEA AR, 99%) were purchased from Sinopharm Chemical Reagent Co., Ltd (Shanghai, China). Cetyltrimethylammonium bromide (CTAB AR 99%) and methanol (AR, 99.5%) were obtained from Tianjin Kemiou Chemical Reagent Co., Ltd (Tianjin, China). 2-methylimidazole (AR, 99%) was purchased from Beijing Bailingwei Technology Co., Ltd (Beijing, China). *Escherichia coli* (ATCC 25922); *Staphylococcus aureus* (ATCC 6538) bacterial strains were obtained *from the China General Microbiological Culture Collection Center.*

### 2.2. Synthesis of Hierarchical ZIF-8

Hierarchical ZIF-8 was synthesized based on a previously reported method with a minor modification [14]. Typically, a solution A was firstly prepared by mixing 1.88 g of Zn(NO_3_)·6H_2_O, 1.27 g CTAB and 2.14 g of histidine in 125 mL of water. Meanwhile, another solution B containing 4.0 g of 2-methylimidazole and 5.0 g of triethylamine was also produced. Then, the solution B was poured into A under magnetic stirring, and the mixture was incubated at 30 °C for 7 h. The solids were separated and washed three times with a methanol and water mixture (v/v = 1, 1), and then dried and denoted as H-ZIF-8.

### 2.3. Loading of Ag Nanoparticles into the H-ZIF-8

Typically, 0.016 g AgNO_3_ (0.06 mmol) was dissolved in 4 mL of methanol and then mixed with 0.5 g of H-ZIF-8. After shaking for 2 h, 2.4 mL of cold sodium borohydride aqueous solution (1 M) was added dropwise into the mixture in an ice bath and shaken for another 12 h at 30 °C. The solid was washed twice with methanol and was separated by centrifugation to obtain a yellow powder. The solid decorated with Ag NPs was named Ag/H-ZIF-8. For comparison, silver nanoparticles were synthesized by reducing AgNO_3_ with NaBH_4_ in the presence of sodium citrate acting as capping agents [15]. Typically, 17 mg of AgNO_3_ and 63 mg of citric acid were dissolved in 500 mL of water under stirring. Then, an excess of freshly prepared aqueous NaBH_4_ solution (2 mM) was added dropwise into the mixture in an ice bath under shaking, the solution immediately turned dark grey. The product was separated by centrifugation at 10,000 rpm and dried under vacuum.

### 2.4. Antibacterial Assay

*Staphylococcus aureus (S. aureus)* and *Bacillus subtilis (B. subtilis)*, as well as *Escherichia coli (E. coli)*, are representatives of gram-positive and gram-negative bacteria, respectively. Therefore, the antibacterial properties of the materials against gram-positive *Staphylococcus aureus (S. aureus)* and *Bacillus subtilis (B. subtilis)*, as well as a gram-negative *Escherichia coli (E. coli)*, were assessed as described below [16,17]. Firstly, all the target bacteria were cultured in Luria-Bertani (LB) medium at 37 °C for 24 h. Then, 400 µL of 10^8^ CFU/mL bacterial suspension was added to 40 mL of LB liquid medium containing different amounts of Ag/H-ZIF-8. The bacteria were cultivated at 37 °C, and the growth rates of, *S. aureus* and, *E. coli* were monitored at 600 nm using a Simadu UV-2450 spectroscopy in a time-dependent manner. For comparison, parallel tests of Ag nanoparticles and ZIF-8 nanoparticles were also conducted by replacing Ag/H-ZIF-8 with hierarchical ZIF-8 and Ag NPs as antibacterial agents at corresponding concentrations. The quantitative antibacterial efficacy was calculated as follows: AE(%) = (1 − B/A) × 100%, where AE(%) is the antibacterial efficacy, A represents the OD_600_ of the untreated bacteria suspension (without nanoparticles), and B is the OD_600_ of the bacteria suspension treated by the nanoparticles, respectively.

Minimal inhibition concentrations (MIC) were measured by a microdilution method [18]. Typically, a suspension of 1 × 10^8^ CFU/mL of bacteria in nutrient broth was prepared as described above. The antibacterial solutions were prepared using serial dilutions of Ag/H-ZIF-8 in concentrations ranging from 0.06 to 0.27 mg/mL and incubated at 37 °C for 24 h. The lowest concentration of tested Ag/H-ZIF-8 that resulted in no visible turbidity was considered as the MIC. 

Furthermore, using, *E. coli* and *Bacillus subtilis* as model bacteria, the agar dilution method was used to further examine the bacterial viability after 24 h treatment with Ag/H-ZIF-8 [16]. Typically, 0–4 mg of Ag/H-ZIF-8 composites were mixed with 40 mL of LB medium under ultrasonic treatment and poured into plates. In addition, 50 µL of the 10^5^ CFU/mL bacterial suspension was then evenly coated onto the agar plate. After 12 h cultivation at 37 °C, photographs were taken to identify the bacteria colonies grown in each plate.

### 2.5. Preparation of Bacterial Samples for SEM 

The preparation of bacterial samples for SEM was conducted according to Ge’s method [19]. After treatment with antibacterial systems, *E. coli* and, *S. aureus* bacteria were collected by centrifugation at 6,000 rpm for 2 min and washed twice with PBS. Then, the bacterial cells were fixed with 2.5% glutaraldehyde overnight at 4 °C. After washing three times with PBS, the cells were dehydrated through graded ethanol solutions (30%, 50%, 75%, 90%, and 100%). Finally, the samples were placed on a silicon wafer and visualized using an S-4800 SEM (Hitachi, Japan).

### 2.6. Cytotoxicity of Ag/H-ZIF-8

Cell viability was measured with CCK-8 according to the manufacturer’s protocol. For the cytotoxicity assay, the Hepatic epithelial cells were cultured in 96-well plates (100 μL, 1 × 10^4^ cells per well) for 24 h. Different concentrations of Ag/H-ZIF-8 nanocomposite (0–150 μg/mL) in culture medium were added to each well and then incubated for 24 h. CCK-8 was used to assess cell viability. The CCK-8 solution (15 μL) was added to each well and incubated with the cells for another 1 h. After thoroughly mixing, the plates were read at 450 nm in an enzyme microplate reader (Synergy-H_1_, USA) for optical density that is directly correlated with the cell quantity. Each result was the average of three wells, and 100% viability was determined from untreated cells.

## 3. Results and Discussion

### 3.1. Characterization of the Synthesized Materials

**Figure 1:**
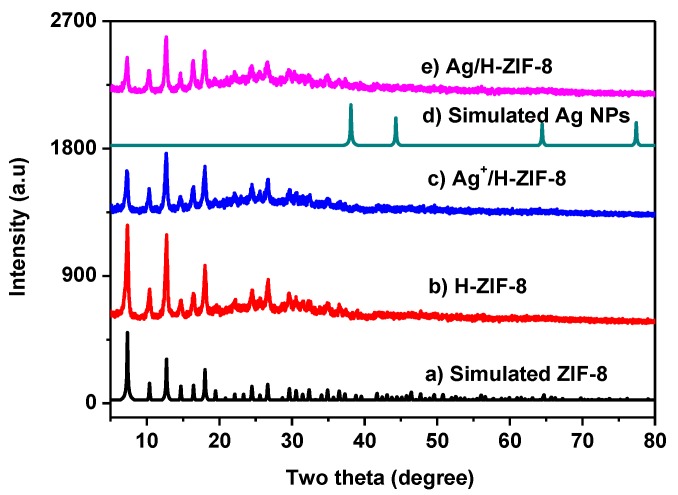
XRD patterns of materials: (**a**) simulated ZIF-8; (**b**) hierarchical ZIF-8; (**c**) Ag^+^/H-ZIF-8; (**d**) simulated Ag Nps; (**e**) Ag/H-ZIF-8.

**Figure 2:**
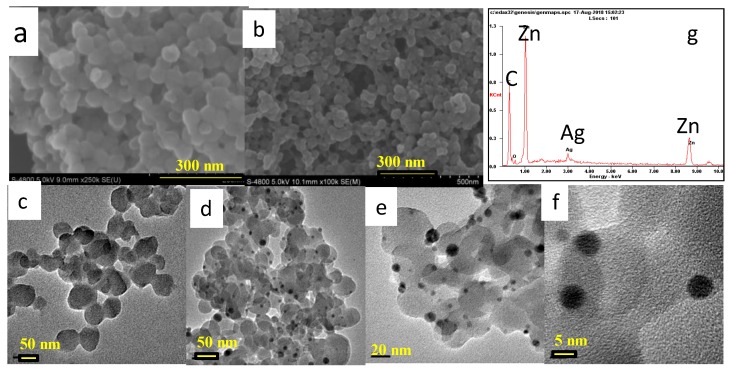
SEM, TEM and SEM-EDX images of materials: SEM images of H-ZIF-8 (**a**) and Ag/H-ZIF-8 (**b**); TEM image of H-ZIF-8 (**c**) and Ag/H-ZIF-8 (**d**–**f**); SEM-EDX of Ag/H-ZIF-8 (**g**).

Figure 1 shows the XRD spectra of Ag/H-ZIF-8 and related materials. Obviously, the typical diffraction peaks of as-synthesized H-ZIF-8 (Figure 1 curve b) matches well with those of simulated (Figure 1 curve a) and reported ones [14,20,21]. Figure 1 curve c and e are the X-ray diffraction patterns of H-ZIF-8 composites decorated with Ag^+^ and Ag NPs, respectively. The typical diffraction peaks assigned to ZIF-8 can all be observed, although the intensity has decreased a little bit. Additionally, no obvious peaks attributed to the silver Nps are observed for the nanocomposite, suggesting the well-dispersed state of the metallic silver in the hierarchical ZIF-8. Therefore, it can be concluded that the as-prepared material has the crystal structure of ZIF-8, and no obvious changes in the ZIF-8 phase after silver deposition were detected. The SEM-EDX measurement (Figure 2e) was further conducted to confirm the existence of Ag and surface composition in the prepared catalyst, which demonstrates that the Ag: Zn atom ratio is about 1, 11.9, and the Ag weight content was calculated to be 3.8%, accordingly.

**Figure 3:**
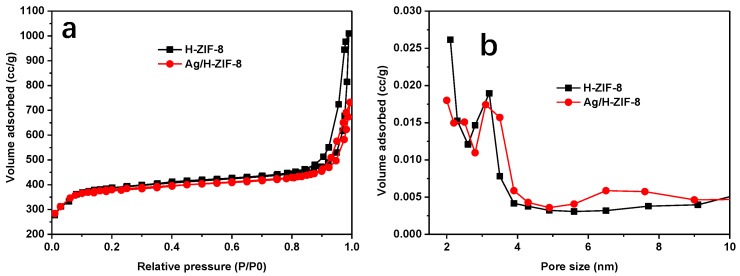
Nitrogen adsorption-desorption isotherms (**a**) and pore-size distributions (**b**) of H-ZIF-8 and Ag/H-ZIF-8.

The SEM and TEM techniques were further used to explore the micro-structure of Ag/H-ZIF-8 nanocomposite. Representative SEM (Figure 2a,b) and TEM (Figure 2c,d) images reveal that the prepared H-ZIF-8 were sphere-like nanoparticles with narrow size distributions. Additionally, mesoporous pores can be clearly observed in the TEM image due to their hierarchical properties. Although no typical diffraction peaks for Ag NPs were detected in the XRD pattern, as expected, highly dispersed Ag NPs in the H-ZIF-8 were detected with the TEM technique, as shown in Figure 2d,e. In addition, nitrogen adsorption experiments were performed to confirm the porous framework of hierarchical ZIF-8 and Ag/H-ZIF-8. As shown in Figure 3a, the obtained isotherms exhibit a mixture of type I and type IV modes, suggesting the coexistence of both micro- and mesoporous cages. The BET specific surface area and the pore volume for the hierarchical ZIF-8 were calculated to be 1520 m^2^/g and 1.56 cm^3^/g, respectively. Furthermore, the average pore diameter of hierarchical ZIF-8 was calculated to be 3.2 nm. The decoration of Ag NPs in the porous material decreased the BET specific surface area and the total pore volume to 1292 m^2^/g and 1.13 cm^3^/g, respectively. The decreased pore volume indicates the encapsulation of Ag NPs inside the pore of the hierarchical ZIF-8.

**Figure 4:**
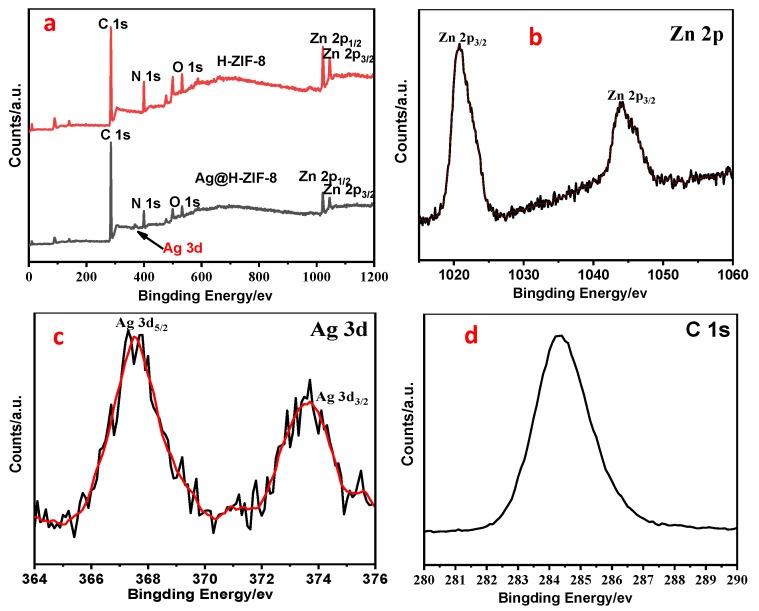
XPS spectra of H-ZIF-8 and Ag/H-ZIF-8. (**a**) Survey; (**b**) Zn 2p; (**c**) Ag 3d; and (**d**) N 1s.

X-ray photoelectron spectroscopy (XPS) measurements were conducted to further explore the surface compositions and oxidation states of the H-ZIF-8 and Ag/H-ZIF-8 composites. Figure 4a shows the survey XPS spectra of the H-ZIF-8 (red line) and Ag/H-ZIF-8 (black line) samples. The results clearly reveal that the Ag/ZIF-8 composite mainly consists of, C, N, O, Zn and Ag atoms, while there are no Ag atoms in the H-ZIF-8, which is in good agreement with their SEM-EDX result. The Zn 2p spectrum of the Ag/H-ZIF-8 sample shown in Figure 4b exhibits two contributions, located at 1020.8 and 1044.0 eV, respectively, which are designated as the binding energy values of Zn 2p_3/2_ and Zn 2p_1/2_, confirming the presence of Zn atoms. Figure 4c presents the binding energy of Ag 3d electrons for Ag/H-ZIF-8, where the peaks at 367.1 and 373.7 eV are ascribed to the Ag 3d_3/2_ and Ag 3d_5/2_ electronic states, respectively. The difference in the binding energy between the above electronic states for Ag 3d electrons is 6.0 eV, which suggests the formation and stabilization of the zero valent chemical state of silver in the Ag^0^/H-ZIF-8 hybrid composite [22,23,24]. Furthermore, the atom percentages of silver and zinc are 0.41% and 3.18%, respectively. Accordingly, the loading content of Ag was calculated to 4.8%, which is a little bit higher than the SEM-EDX result.

### 3.2. Antibacterial Activity

**Figure 5:**
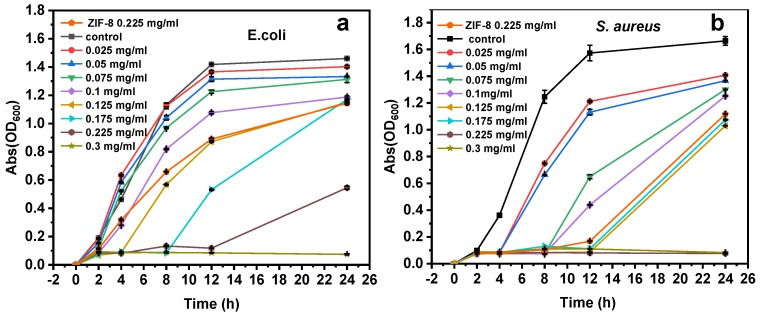
Growth curves of (**a**) *E. coli* and (**b**) *S. aureus* inoculated with different concentrations of Ag/H-ZIF-8.

**Figure 6:**
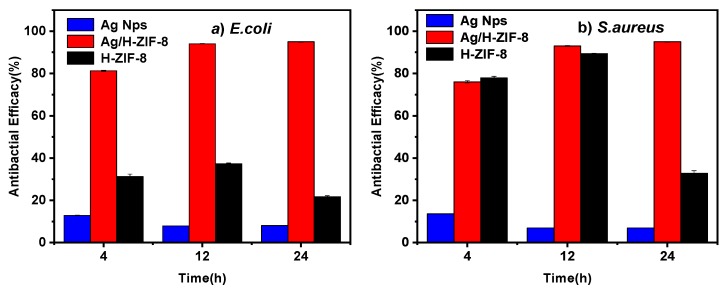
Antimicrobial efficiencies of Ag, H-ZIF-8 and Ag/H-ZIF-8 for (**a**) *E. Coli* and (**b**) *S. aureus* calculated at 4, 12 and 24 h, respectively.

*Escherichia coli (E. coli)* and *Staphylococcus aureus (S. aureus)* are representatives of gram-negative and gram-positive bacteria, respectively. Therefore, the antibacterial actions of Ag/H-ZIF-8 nanocomposite were firstly investigated against both, *E. coli* and, *S. aureus* in the liquid. For the kinetic tests, the relationships between the concentrations of the Ag/ZIF-8 and bactericidal activities were investigated, and the results were assayed by measuring optical density at 600 nm. As shown in Figure 5 and Figure 6, in the control tests, the pure H-ZIF-8 show little antibacterial effect on both the gram-positive bacteria, *S. aureus* and gram-negative, *E. coli* strains. Many studies have shown that the released zinc ions from Zn-based nanomaterials and/or the attachment of nanoparticles or their aggregates to the organism were responsible for their nanotoxicity [25,26,27,28,29]. The Ag NP-decorated H-ZIF-8 progressively inhibited the growth of the organism and showed a significantly enhanced antibacterial property compared to the same dose of pure H-ZIF-8. Moreover, using the same dosage of Ag NPs (the TEM image is shown in Figure S1) prepared without stabilized ligand or supports led to a much lower inhibition effect on both gram-negative and gram-positive bacterial strains (Figure S3), probably due to their aggregations in the liquid. Obviously, the nanocomposite demonstrated much higher antibacterial efficiency than the single pure ZIF-8 and Ag Nps (shown in Figure 6), indicating that the copresence of Ag Nps and ZIF-8 provided a synergistic effect on its antibacterial activity [16,25]. As reported by Guo et al., they also observed that the core-shell Ag@ZIF-8 nanowires exhibited much higher antibacterial activities than pure Ag nanowires and ZIF-8 [16]. They explained that the presence of Ag@ZIF-8 not only achieves sustained release of silver ions, but also stabilizes the silver core against aggregation. Therefore, the composite exhibited the best antibacterial activities. More interestingly, gram-positive bacterial strains are more susceptible to Ag/H-ZIF-8 than gram-negative bacteria. For example, the growth of bacterial colonies is completely prevented for *S. aureus* with a concentration of 0.225 mg/mL. Meanwhile, the concentration for *E. coli* was up to 0.30 mg/mL. Clearly, Ag/H-ZIF-8 shows stronger antibacterial efficiency for gram-positive *S. aureus* than gram-negative *E*. *coli.* Our data are in good agreement with studies carried out by other groups [16,30,31,32]. 

The minimal inhibition concentration can quantitatively evaluate the antibacterial activities. The MIC is the minimum concentration of an antibacterial agent needed to completely inhibit bacterial growth within 12 h or 24 h. The gram-negative, *E. coli* and gram-positive, *S. aureus* bacteria are usually used in antibacterial assays to evaluate antibacterial activity. The antibacterial solutions were prepared using serial dilutions of Ag/H-ZIF-8 in concentrations ranging from 0.06 to 0.27 mg/mL and incubated at 37 °C for 24 h. The lowest concentration of each tested Ag/H-ZIF-8 that resulted in no visible turbidity was considered as the MIC. As shown in Figure S4a, in the case of, *E. coli*, the MIC of Ag/H-ZIF-8 is determined to 0.25 mg/mL for 24 h. For, *S. aureus*, the MIC of Ag/H-ZIF-8 is determined to be 0.17 mg/mL for 24 h, respectively (shown in Figure S4b). Clearly, Ag/H-ZIF-8 shows stronger antibacterial efficiency for gram-positive, *S. aureus* than gram-negative, *E. coli*, which is consistent with the OD_600_ measurement results. We had known that gram-positive bacteria have a thicker peptidoglycan layer than the gram-negative bacteria. However, gram-negatives possess a relatively impermeable lipid membrane. Therefore, gram-positive bacteria are more receptive to certain chemical agents than the gram-negatives.

**Figure 7:**
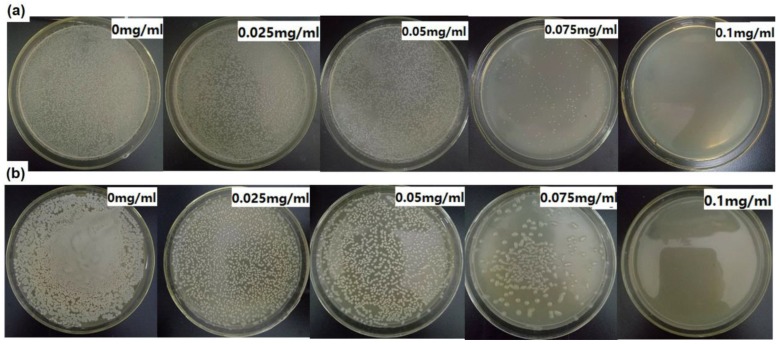
*E. coli* (**a**) and *B. subtilis* (**b**) grown on agar plates with various concentrations of Ag/H-ZIF-8.

Encouraged by the above results, growth on agar plates was further used to detect the antibacterial activity of Ag/H-ZIF-8 composite against pathogenic bacteria using, *E. coli* and, *B*. *subtilis* as models. Interestingly, results shown in Figure 7 showed a similar trend as that found in the liquid-growth experiments. Various concentration of Ag/H-ZIF-8 was loaded into the agar plate and efficient inhibition phenomenon was observed at higher concentrations. For example, the growth of bacterial colonies could be both completely inhibited with 0.1 mg/mL of Ag/H-ZIF-8. Comparatively, no inhibition phenomenon was observed using the same dose of Ag Nps. Furthermore, a more than 6 times higher dosage of Ag Nps should be loaded if we want to get the similar inhibition effect (Figure S5). All results above demonstrate that the Ag/H-ZIF-8 composite have outstanding antibacterial activity against both positive and negative bacteria.

**Figure 8:**
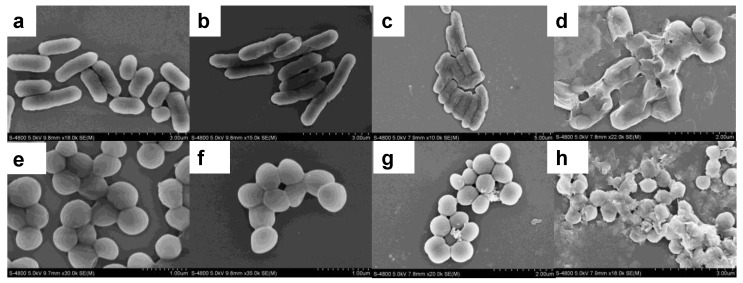
SEM images of (**a**) *E. coli*; (**b**) *E. coli* treated with H-ZIF-8; (**c**) *E. coli* treated with Ag Nps; (**d**) *E. coli* treated with Ag/H-ZIF-8; (**e**) *S. aureus*; (**f**) *S. aureus* treated with H-ZIF-8; (**g**) *S. aureus* treated with Ag Nps; (**h**) *S. aureus* treated with Ag/H-ZIF-8.

To investigate the changes of bacterial morphology induced by the antibacterial system, SEM was used to observe, *E. coli* and, *S. aureus* before and after treatment (Figure 8). As shown in Figure 8a, untreated, *E. coli* cells were typically rod-shaped, with smooth and intact cell walls. SEM images shown in Figure 8d indicated that the, *E. coli* cells completely lost their cellular integrity after exposure to Ag/H-ZIF-8 dispersions, since Ag^+^ could oxidize the lipid membrane and destroy the bacterial membranes. As for *S. aureus* bacteria, the results of SEM experiments were similar to those of *E. coli* cells (Figure 8e–h). In the control group, the, *S. aureus* cells were sphere-shaped and smooth; After treatment with Ag/H-ZIF-8 as antibacterial agent, the, *S. aureus* cells became seriously rough and damaged. The results were consistent with those recorded on optical spectroscopy, which suggested that Ag/H-ZIF-8 were highly efficient for bacteria killing.

### 3.3. Cytotoxicity Assay

**Figure 9:**
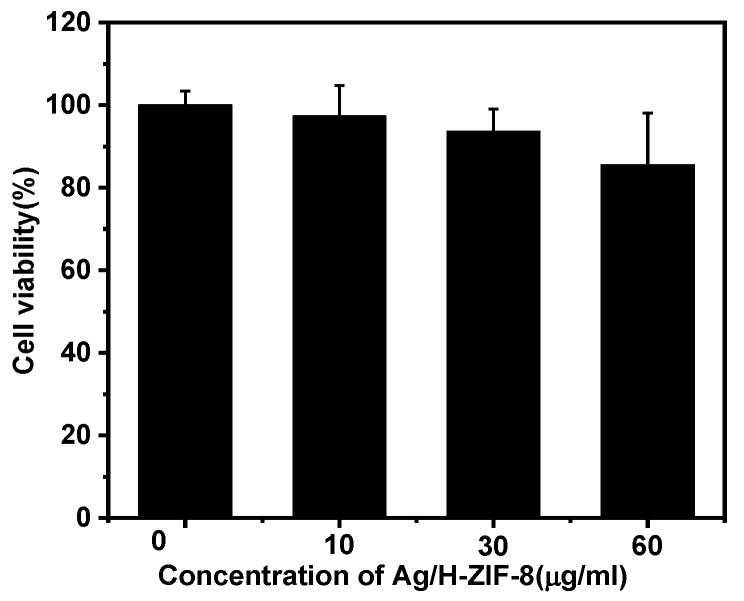
The survival rate of different concentrations of Ag/H-ZIF-8 co-cultured with Hepatic epithelial cells.

The above results sufficiently indicate that Ag/H-ZIF-8 can be of great benefit in antibacterial disinfection. Therefore, the biosafety of this nanomaterial should be taken into accounts. Herein, the cytotoxicity of the Ag/H-ZIF-8 on Hepatic epithelial cells was evaluated. As a result, as shown in Figure 9, no obvious toxicity was observed on the cell viability after 24 h of exposure to the Ag/H-ZIF-8 nanocomposite, even at the concentration of Ag/H-ZIF-8 up to 60 μg/mL. Overall, this result indicates that the Ag/H-ZIF-8 nanocomposite has low cytotoxicity and good biocompatibility. All results above demonstrated the great potential of Ag/H-ZIF-8 composite in practical antibacterial applications, and that its delivery can be implemented by using fibrous materials which can be made by many methods [33,34,35,36].

## 4. Conclusions

In summary, well-defined metallic Ag NP-decorated hierarchical ZIF-8 was fabricated, and its structure and morphology were systematically characterized by a series of techniques. Furthermore, its antibacterial properties were assessed using *S. aureus*, *B. subtilis* and *E. coli* as model strains. The hierarchical structures of the ZIF-8 and corresponding metallic Ag NPs decorated materials were confirmed by both the TEM images and N_2_ sorption experiments. Moreover, the Ag NPs were uniformly dispersed and has a narrow size distribution. The Ag/H-ZIF-8 composite showed outstanding inhibition effect on the growth of both gram-positive (*B. subtilis and, S. aureus*) and gram-negative bacteria (*E. coli*), which can be attributed to the synergistic effect between the H-ZIF-8 and Ag Nps. Furthermore. Ag/H-ZIF-8 demonstrated good biocompatibility. All results above demonstrate the great potentials of Ag/H-ZIF-8 composites in practical antibacterial applications.

## Supplementary Materials

The following are available online at https://www.mdpi.com/2079-4991/9/11/1579/s1, Figure S1: TEM image of Ag NPs; Figure S2: FT-IR spectra of H-ZIF-8 and Ag/H-ZIF-8; Figure S3: Growth curves of (a) *S. aureus* and (b) *E. coli* inoculated with different concentrations of Ag Nps, respectively; Figure S4: MIC of *E. coli* (a) and *S. aureus*(b) inoculated with different concentrations of Ag/H-ZIF-8 for 24 h, respectively; Figure S5: *E. coli* (a) and *B. subtilis* (b) grown on agar plates using various concentrations of Ag NPs.
