# Supplementary material for: Ag/H-ZIF-8 Nanocomposite as an Effective Antibacterial Agent Against Pathogenic Bacteria
Source: Nanomaterials (Basel). 2019 Nov 7;9(11):1579. doi: 10.3390/nano9111579 (PMC6915408; doi:10.3390/nano9111579)
Supplement: Supplementary file 1 [file nanomaterials-09-01579-s001.pdf]

Electronic Supplementary information

for

Ag/H-ZIF-8 Nanocomposite as an Effective Antibacterial Agent Against Pathogenic Bacteria

Yanmei Zhang[1,2*], Xin Zhang[1,2], Jie Song[1,2], Liming Jin[1,2], Xiaotong Wang[1,2], Chunshan Quan[1,2*]

[1]College of Life Science, Dalian Minzu University, Economical and Technological Development Zone, Dalian, 116600, China; [2]Key Laboratory of Biotechnology and Bioresources Utilization (Dalian Minzu University), Ministry of Education, China

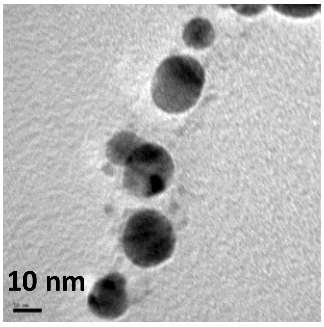

Fig. S1 TEM image of Ag NPs

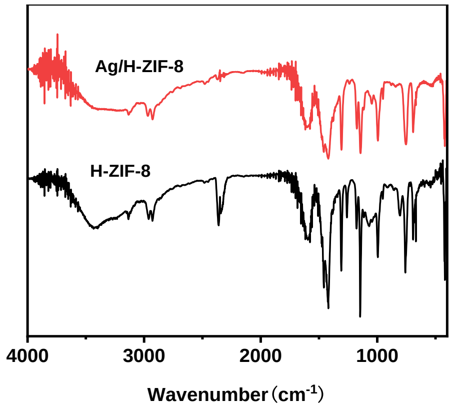

Fig. S2 FT-IR spetra of H-ZIF-8 and Ag/H-ZIF-8

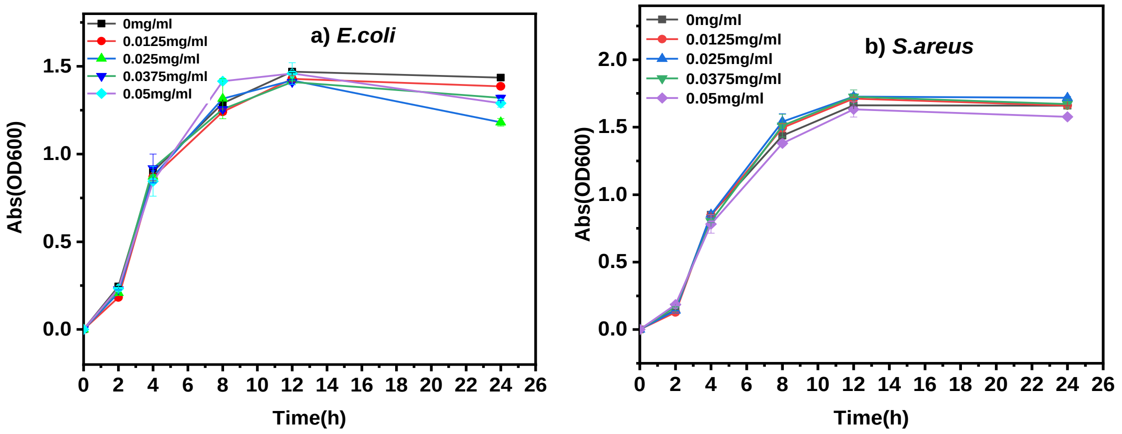

Fig. S3 Growth curves of (a) *S. Aureus* and (b) *E. coli* inoculated with different concentrations of Ag Nps.

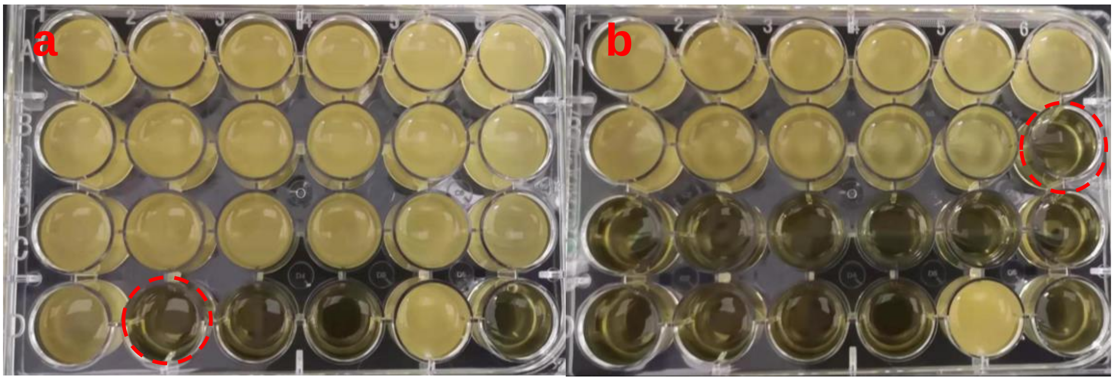

Fig. S4 MIC of *S. aureus*(a) and *E. coli* (b) inculated with different concentrations of Ag/H-ZIF-8 Nps.

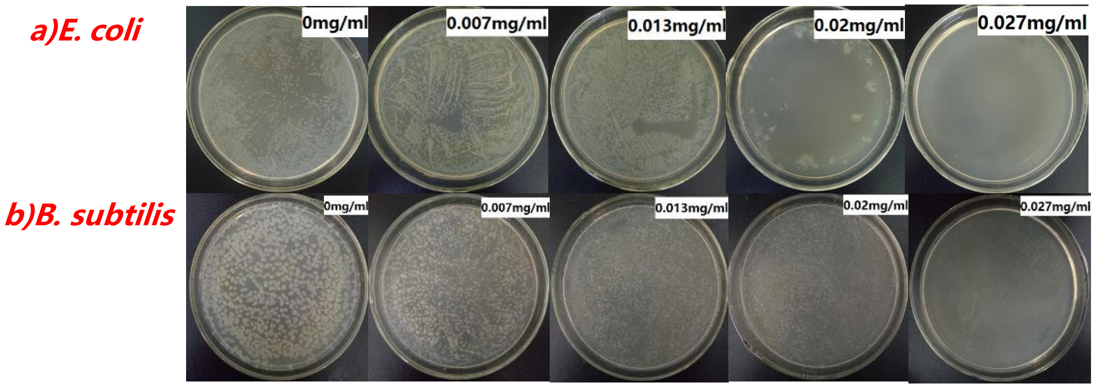

Fig. S5 *E. coli* (a) and *Bacillus subtilis*(b) grown on agar plates using various concentrations of Ag NPs.
